# MOSY: A method for synthetic opinions to yield a robust fuzzy expert system

**DOI:** 10.1016/j.mex.2023.102112

**Published:** 2023-03-08

**Authors:** N. Gnaneshwara, B.V. Vijay

**Affiliations:** Department of Aerospace Engineering, M S Ramaiah University of Applied Sciences, 470-P, Peenya 4th Phase, Peenya Industrial Area, Bengaluru, Karnataka 560058, India

**Keywords:** Synthetic opinion, Fuzzy expert system, Optimum rule weights, Expert-character, Decision-making, MOSY, Method for Synthetic Opinions

## Abstract

In many domains, decision-making is challenging, as experts are often limited in availability. However, without a sufficient number of expert opinions, the associated solutions would not be robust. Motivated by this, MOSY, a Method for SYnthetic Opinions has been developed to produce a robust Fuzzy Expert System (*FES*) by specifying $N_{sr}$, the number of (synthetic) experts per rule. For every one of these “synthetic experts”, MOSY produces an opinion from a normal distribution characteristic of a human expert. Correspondingly, the *FES* is used to produce an opinion from an antecedent vector whose elements are sampled from a uniform distribution. Synthetic and human opinion vectors, resulting from all rules and number of experts per rule, are driven to agree through optimization of weights associated with the fuzzy rules. The weight-optimized MOSY was tested against sets of human expert opinions in two distinct domains, namely, an industrial development project (*IDP*) and passenger car performance (*PCP*). Results showed that the synthetic and human expert opinions correlated between 91.4% and 98.0% on an average over $5\leq N_{sr}\leq 250$, across five outcomes of the *IDP*. Likewise, for *PCP*, respective correlations varied between 85.6% and 90.8% for $10\leq N_{sr}\leq 150$ across the two performance measures. These strong correlations indicate that MOSY is capable of producing synthetic opinions to yield a robust *FES* where sufficient human experts are not available.

• This method, known as MOSY, generates synthetic expert opinions to achieve robustness in an *FES*.
• MOSY was validated against sets of human expert opinions in two distinct domains.
• Strong correlations were observed between the synthetic and human expert opinions.

## Graphical abstract

**Figure:**
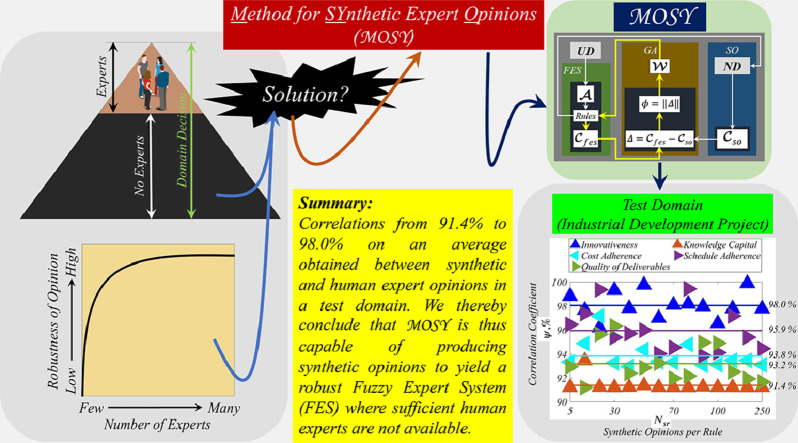

Specifications table

**Table:** 

Subject area:	*Economics and Finance*
More specific subject area:	*Expert Systems*
Name of your method:	MOSY, *Method for Synthetic Opinions*
Name and reference of original method:	*N/A*
Resource availability:	*MATLAB*

**Table:** 

**Mnemonics and acronyms**	

B	big
EL	elderly
E	excellent
*FES*	fuzzy expert system
F	fair
*GA*	genetic algorithm
G	good
HE	human experts
H	high
L	low
M	matured
MOSY	method for synthetic opinions
*ND*	normal distribution
S	small
*SO*	synthetic opinions
*UD*	uniform distribution
VB	very big
VG	very good
VH	very high
VL	very low
VS	very small
VY	very young
Y	young
**Symbols**	
A	array of antecedent vectors
$A_{\mathrm{dir}}$	vector of direct background factors
$A_{cpd}$	vector of computed background factors
$A_{ctg}$	category-travel profile vector
$C_{fes}$	array of consequent vectors from *FES*
$C_{so}$	array of consequent vectors from synthetic opinions
$d_{T}$	total distance travelled
d	vector of distances
f(x)	“normal” probability density function
$N_{bf}$	total number of background factors (antecedents)
$N_{ru}$	number of rules
$N_{so}$	total number of synthetic opinions
$N_{sr}$	number of synthetic opinions per rule
$N_{tp}$	total number of places considered
$N_{yrs}$	age, years
$N_{fs}$	number of family members
$P_{T}$	truth vector of places visited
$t_{opt}$	CPU time for optimisation
$t_{xp}$	optimisation time per synthetic opinion
W	vector of rule weights across rules
$W_{sr}$	vector of rule weights across $N_{sr}$
$W_{ctg}$	category-weights matrix
$x_{\min},x_{\max}$	support of membership function
Δ	array of differences between $C_{fes}$ and $C_{so}$
λ(x)	membership function
ϕ	Euclidean norm of Δ
ψ	correlations

## Method details

***Idea:*** To introduce the idea underlying the method presented here, consider a simple problem of measuring $x_{tru}$, the true length of a bar. Trained metrologists (experts) independently measuring $x_{tru}$ would report values $x_{mea}$ that are scattered in the form of a classical normal distribution (*ND*) about $x_{tru}$. Thus, from a sufficient number of independent expert measurements about $x_{tru}$, one could obtain a reliable estimate of $x_{tru}$. Observed another way, the degree of expertise is characterized by the extent of deviation of $x_{mea}$ from the mean or in general, the centre of truth ($x_{tru}$ in the above case). Drawing from this analog, if one were to consider a *FES* [1] composed of a fixed set of antecedents, one could “synthetically” produce the associated “expert-generated” consequent ($x_{mea}$) by sampling from an *ND* adopting $x_{tru}$ as mean and standard deviation that reflects the degree of expertise.

**Fig. 1:**
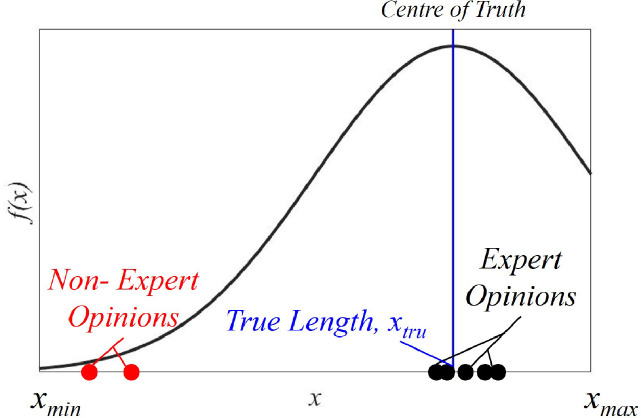
Expert and non-expert opinions about a true length.

Fig. 1 illustrates the above idea comparing expert and non- expert opinions over the true length, $x_{tru}$. While expert opinions (points in black) are most likely to **cluster** around $x_{tru}$, such opined measurements would be far away from $x_{tru}$ when reported by non-experts (points in red). In Fig. 1, $x_{\min}$ and $x_{\max}$ stand for the smallest- and largest lengths measurable by the metrologists.

Earlier, Yang et al. [2] have demonstrated a similar concept of generating virtual samples from a Gaussian distribution to produce training sets for use in neural networks. Although the present idea also relies on such sampling, the originality of our method is the novel implementation into an *FES*.

***MOSY***

**Fig. 2:**
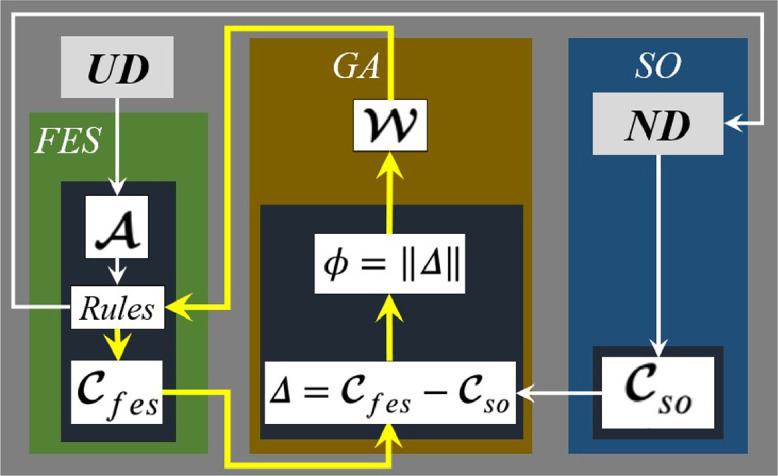
MOSY framework.

***Working:*** Building on the idea described above, MOSY, a Method for SYnthetic Opinions was developed and described here. As shown in Fig. 2, MOSY is composed of three modules: *FES*, a genetic algorithm (*GA*) [3] for rule weight optimization and *SO*, a module for synthetic opinion generation. The *FES* is constructed based on the knowledge of only one human expert.

**Fig. 3:**
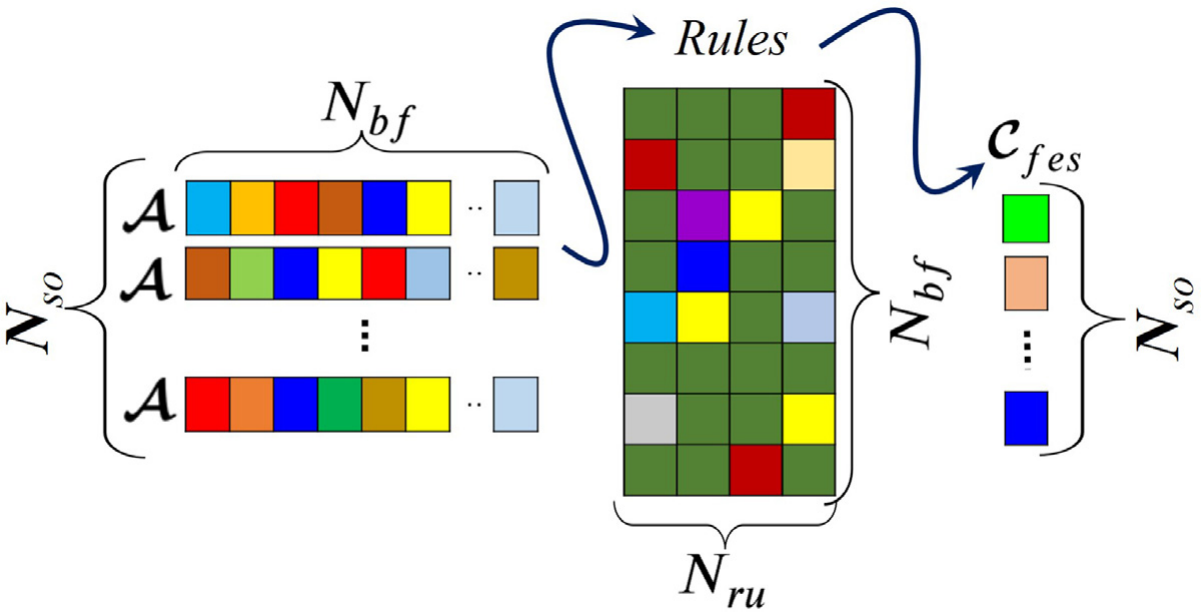
Generation of $C_{fes}$ from $N_{so}$ vectors A.

The *SO* module, a key constituent of MOSY, produces a scalar sample from the *ND characteristic of an expert opinion*, as discussed in 'Method details'. On adopting $N_{sr}$, which is the number of (synthetic) experts for each rule of *FES, SO* is used to generate a total of $N_{so}=N_{ru}\times N_{sr}$ samples, forming the vector of synthetic opinions $C_{so}$ in Fig. 2. Correspondingly, on the *FES* side, against each of the $N_{so}$ samples, an antecedent vector A is generated with elements sampled from a uniform distribution (*UD*). Such *UD* samples lie within the support of the membership functions associated with A. For each A, a consequent is produced through the respective rules matrix of size $N_{ru}\times N_{bf}$ from the standard Mamdani inference process shown in Fig. 3, leading to $C_{fes}$, the vector of $N_{so}$ consequents from as many antecedent vectors A.

**Fig. 4:**
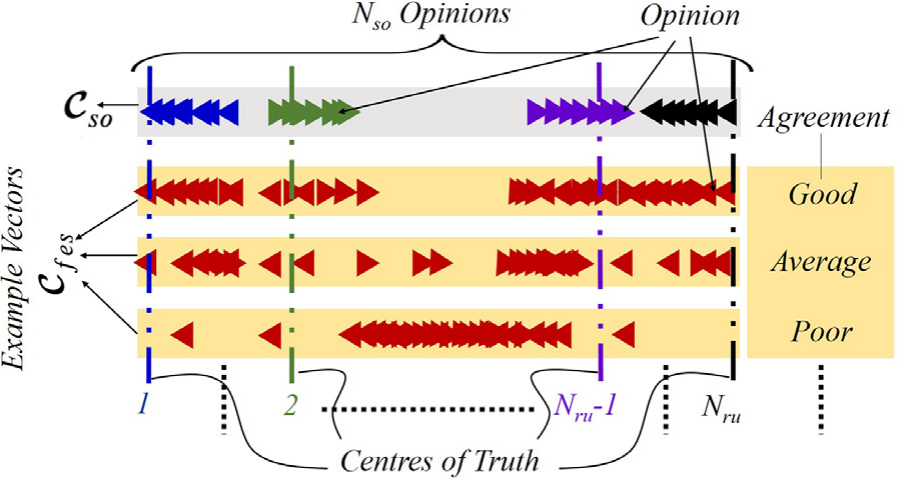
Rationale of MOSY with example agreements between $C_{fes}$ and $C_{so}$.

***Rationale:***Fig. 4 illustrates the rationale of MOSY, according to which $C_{so}$ represents the true opinions and with increasing $N_{sr}$, $C_{fes}$ can be driven to increasingly agree with $C_{so}$. More precisely, $C_{fes}$ character is determined both by character of the underlying A as well as the W. At the same time, as Fig. 4 shows, $C_{so}$ would possess a multi-cluster character, with each cluster forming about a *center of truth* associated with each of the $N_{ru}$ rules. Also, as the figure shows, $C_{fes}$ and $C_{so}$ would have a good agreement when opinions from the former closely pattern those from the latter. On the other hand, agreement would be average to poor for moderate to contrasting opinion patterns between the two vectors. Accordingly, to maximize agreement between the $C_{fes}$ and $C_{so}$, we adopt Euclidean norm of the difference between $C_{fes}$ and $C_{so}$ as the objective ϕ, to be minimized by search, across the W space, as given by (1).

(1) $$\phi \left(W\right)=∥C_{fes}-C_{so}∥$$

The *GA* generations continue until ϕ falls below a specified tolerance. The above process leads to a robust *FES* with weights optimized for maximum agreement between human ($C_{fes}$) and synthetic expert opinions ($C_{so}$). Robustness is attributable to the fact that many (synthetic) expert opinions shape the rule weights.

***Related work:*** Synthetic data generation methods have been considered across distinct domains such as synthetic time series generation [4], synthetic electronic medical records (of a fairly critical nature) [5], vehicle image domain randomization [6], use of Kriging and Radial Basis Functions in Bayesian networks [7] and synthetic keystroke dynamics [8]. In these works, the common motivation was largely the need to compensate for the lack of real-world data.

Decision-making is a key step in all business domains. In turn, domain expertise is the key to decision-making. Many niche domains lack sufficient expertise, either because of an insufficient number of experts or of knowledge itself. While an accurate *FES* could possibly compensate for this, the challenge lies in achieving accuracy as well as robustness of the resulting opinion(s). In our effort to address this challenge, ideas have been drawn from the authors’ own expertise as well as sources such as the excellent treatise by Ericsson et al. [9]. In particular, the present method’s idea of sampling about a center of truth could be seen to correlate well with the findings of Salas et al. [10], who noted that experts are characterised by a *shared mental model* in their domain.

## Method validations

To validate MOSY, we assessed the agreement ψ:(0≤ψ≤1) between vectors of real-world opinions derived from questionnaire responses from a set of human experts, $C_{he}$, and $C_{fes}$ from the *FES*, as defined by (2). Each questionnaire has two response components: 1. a set of background factor ratings and 2. the expert opinion rating. The background factor ratings serve to determine $C_{fes}$ in validation whereas $C_{he}$ is the vector of expert opinion ratings. Precisely, from a single expert’s vector of background factor ratings, a de-fuzzified outcome (consequent) is produced from the *FES*, thereby amounting to a vector $C_{fes}$ from background factor ratings from all experts.

(2) $$\psi =1-\left[\frac{∥C_{he}∥-∥C_{fes}∥}{∥C_{he}∥}\right]$$

MOSY is validated in two distinct real-world domains: an *Industrial Development Project (IDP)* and *Passenger Car Performance (PCP)*. All computations were performed using MATLAB 2021b with fuzzy logic and optimization toolboxes on an HP Core i5 8th Gen notebook.

***IDP***

***Significance:*** In-house development projects play a major role in the growth of all industries. Outcomes in any project’s domain are influenced by various background factors. As development involves significant time and capital, it is natural for management to seek prior estimates of outcome measures against the background factors as accurately as possible. Although domain experts might be able to provide such estimates, their numbers are more limited than not. One such domain is that of aerospace control systems, where expertise is highly limited, as discussed next.

$C_{he}:$ To obtain $C_{he}$, the questionnaire in Table A.1 was constructed. The questionnaire was designed to seek ratings on the influence of 12 background factors and 5 measures of outcomes in development projects. Precisely, expert opinions were sought on a scale of 1 (very low impact) to 5 (very high impact) for *Innovativeness (IN), Knowledge Capital (KC), Cost Adherence (CA), Schedule Adherence (SA)* and *Quality of Deliverables (QD)*.

**Table 1:** $W_{k}^{sr}$ vs. Rule k, *IDP*.

**No.**	**Outcome measure**	**Rule,**k			
		**1**	**2**	**3**	**4**
1	*IN*	0.4400	0.6469	0.5778	1.0000
2	*KC*	0.0002	0.0001	1.0000	0.8736
3	*CA*	0.3191	0.5412	1.0000	0.5608
4	*SA*	0.8869	0.1274	1.0000	0.4921
5	*QD*	0.2595	0.3791	1.0000	0.8655

**Fig. 5:**
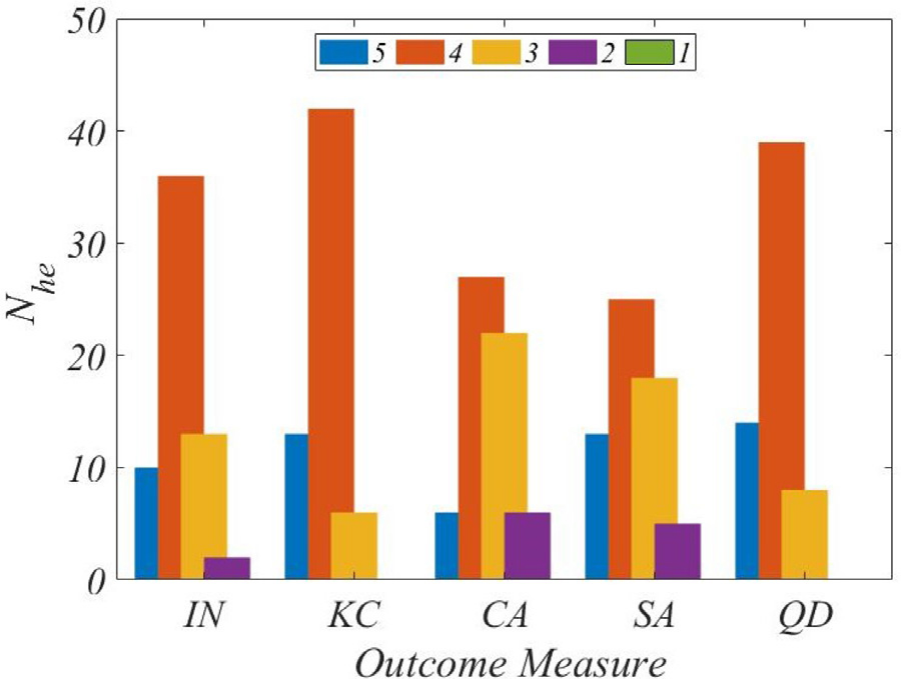
$N_{he}$ distributions, *IDP*.

Of 105 experts who were sent the questionnaire in Table A.1, opinion responses were received from 61 experts, which is thus the length of $C_{he}$. These human opinions would need to be of “expert character” in order for validation to be meaningful, and such character is “normal-like” as reasoned in 'Method details'. Accordingly, we first set out to examine the frequency distribution of the responses to the questionnaire, as shown in Fig. 5 in which the numbers of responses, $N_{he}$, are plotted for *IN, KC, CA, SA,* and *QD*.

In Fig. 5, a “dominant-high-frequency” can be noted for each of the five outcome measures. For instance, in the case of *KC* and *QD*, a rating of 4 is shared across as many as 42 and 39 responses respectively (69% and 64%). Because such “dominant-high-frequency” is “normal-characteristic”, we could opine that the received responses collectively reflect expertise. Indeed, at a 5% significance level, the $\chi^{2}$ goodness-of-fit test confirms the “normal” character of all the five distributions.

**Fig. A.1:**
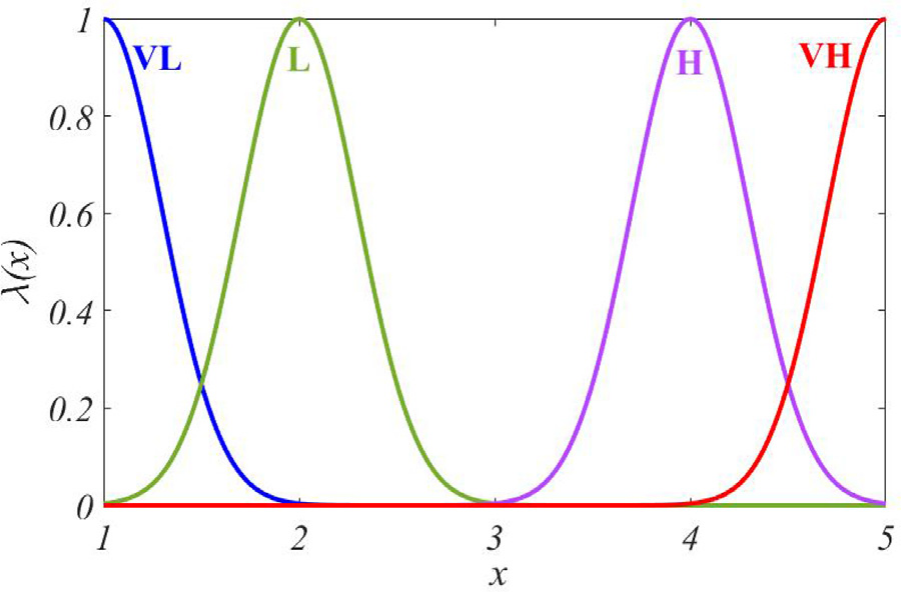
Membership Functions, *IDP*.

$C_{fes}:$Table A.3 gives the rules matrix used to produce each element of $C_{fes}$. Four memberships are adopted for each fuzzy variable (antecedent/ consequent): VH (very high), H (high), L (low) and VL (very low). All membership functions are Gaussian, with a common support $\left(x_{\min},x_{\max}\right)\equiv \left(1,5\right)$ as seen in Fig. A.1. Functions VL, L, H and VH have peaks at x=1,2,4 and 5 respectively. Importantly, this study does not consider a membership function corresponding to what would be termed an “average”, with a peak at x=3. This is because in the authors’ belief, the opinion of an “average” character would be of poorer value than any of VL, L, H and VH.

**Fig. A.2:**
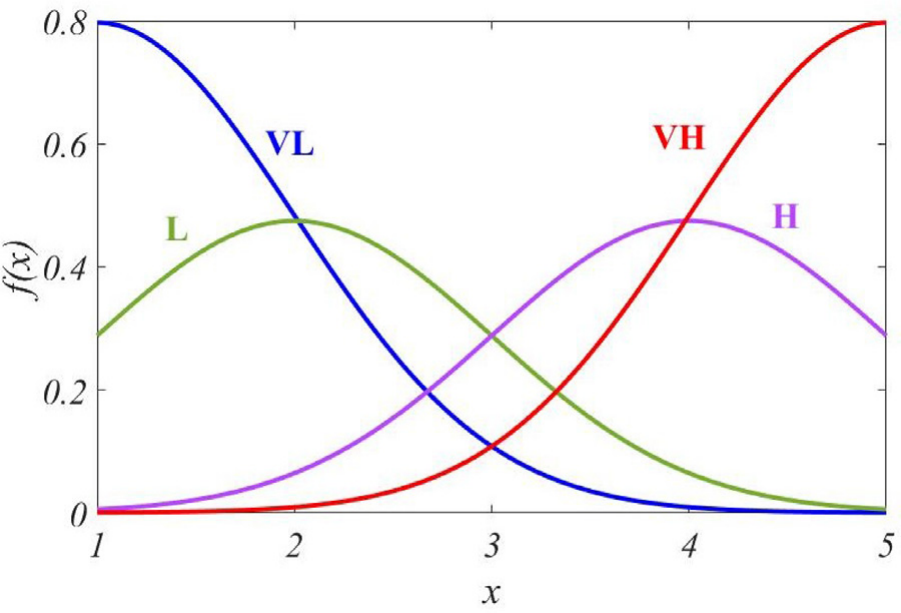
Normal Distributions, *IDP*.

$C_{so}:$$C_{so}$ is derived by sampling from the normal distributions shown in Fig. A.2. These distributions are associated with the four opinions VH, H, L and VL in Table A.3. The *centers of truth* are located at x=1,2,4 and 5 for VL, L, H and VH respectively. All four distributions are normalized to yield a unit area over 1≤x≤5.

**Fig. 6:**
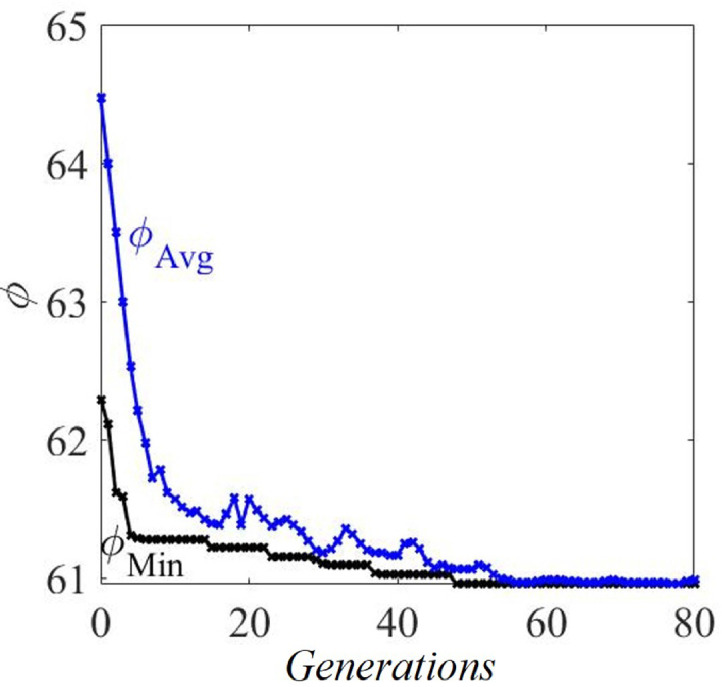
ϕ vs. *Generation, IDP.*

***Optimum rule weights:** GA* was run adopting a population size of 500, crossover fraction of 0.8, and convergence tolerance of $∥\phi ∥\approx 10^{-6}$. To assess the influence of $N_{sr}$, the runs were made for a total of 14 increasing values of $N_{sr}=5,10,20,30,40,50,60,70,80,90,100,125,150$ and 250. For each $N_{sr}$, the optimized W and correlation coefficient ψ (Refer (2)) were obtained. Figure 6 shows the clear convergence of ϕ with generations in the example case of $N_{sr}=250$. Finally, from the optimized W for each $N_{sr}$, the metric $W_{k}^{sr}$ is computed, defined by

(3) $$W_{k}^{sr}=\left[\frac{W_{k}^{{}^{\prime }}.W_{k}}{max(W_{k}^{{}^{\prime }}.W_{k})}\right],k=1,2,\ldots ,N_{ru}$$

where $W_{k}$ is of length $N_{sr}$ and represents the kth elements of the optimized W across all the $N_{sr}$. $W_{k}^{sr}$ would show the relative significance of the kth of the $N_{ru}$ rules.

Table 1 gives the $W_{k}^{sr}$ for each of the four rules, k=1,2,3, and 4, in the IDP for each of the five outcomes. From Table 1, in the case of *KC*, for instance, we see that rules 3 and 4 ($W_{k}^{sr}=1.0$ and 0.8736, respectively) are far more significant than rules 1 and 2 ($W_{k}^{sr}=0.0002$ and 0.0001, respectively).

**Fig. 7:**
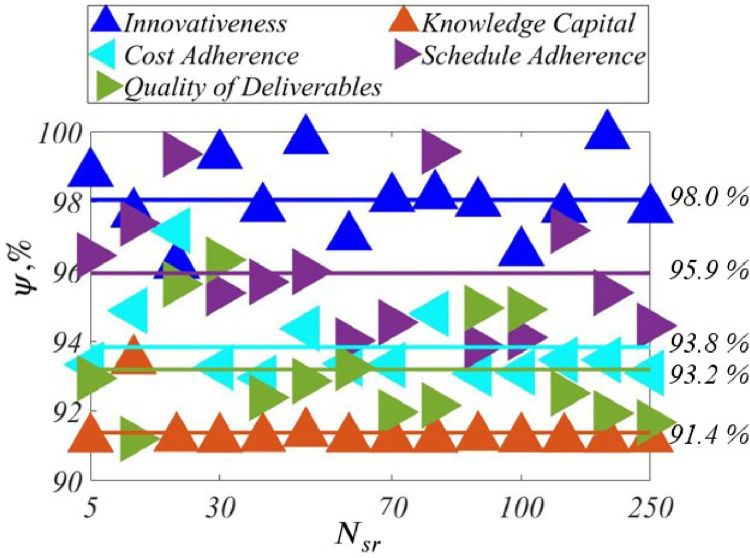
ψ vs. $N_{sr}$, *IDP*.

***Correlations**,*ψ**:**Figure 7 shows the correlations, ψ, across $N_{sr}$ for all the five outcome measures of the IDP. We see that ψ is relatively high, going from ψ=91.4% to ψ=98.0% on an average across the $N_{sr}$. This goes to show that MOSY is effective in leading to high agreement between synthetic and human expert opinions.

***PCP***

***Significance:*** This domain is of significance to both prospective passenger car buyers and car sales executives. For instance, in a typical car dealer’s showroom, a prospective customer would seek the vehicle model most suited to his/her usage needs. Usage needs would vary widely across age groups and travel cultures. For instance, a young customer might opt for a high hauling power if they often travel across mountainous places. Similarly, for heavy usage in commuting within a city, a high fuel economy might be desired by a middle-aged customer with a moderate family size. It is reasonable to assume that both the buyer and sales executive can only have a limited sense of the most appropriate vehicle model, given such complexity of the background and needs. In other words, expertise is typically limited to the “domain” of car choice against need. Accordingly, we considered this domain to test MOSY.

**Fig. 8:**
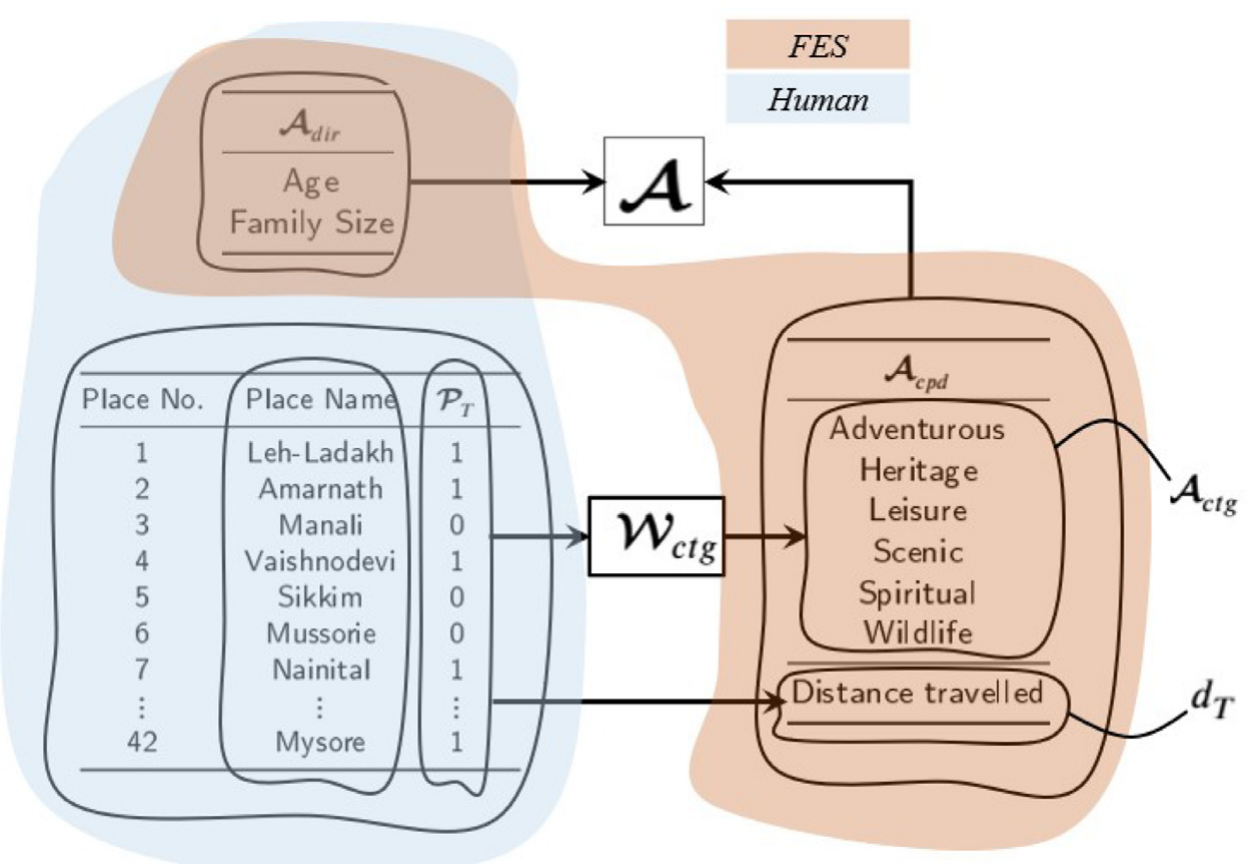
A, *PCP*.

$C_{he}$**:**    To obtain $C_{he}$, the questionnaire in Table A.2 was sent to a set of expert users of passenger cars, seeking opinions on a scale of 1 (fair) to 4 (excellent) for a set of two performance parameters, namely, *Hauling Power (HP)* and *Fuel Economy (FE)*. These two parameters pertain to a set of nine associated background factors A, out of which only two ($A_{dir}$), namely, age and family size, are directly obtained from the expert user inputs unlike all the background factors were directly obtained in case 1 (*IDP*). The seven background factors $A_{cpd}$ were computed by adopting the process illustrated in Fig. 8. $A_{dir}$ and $A_{cpd}$ are defined by (4).

(4) $$A_{dir}=\left[\begin{array}{c} N_{yrs}\\ N_{fs} \end{array}\right];\;A_{cpd}=\left[\begin{array}{c} A_{ctg}\\ d_{T} \end{array}\right]$$

where $A_{ctg}$ is defined as

(5) $$A_{ctg}=P_{T}^{{}^{\prime }}.W_{ctg}$$

where $P_{T}$ is the truth vector for places visited, with any element equal to 1 for a place visited and 0 otherwise and $W_{ctg}$ is the category-weights matrix given in Table A.5. Specifically, for each category, the $W_{ctg}$ value is a fraction in the (0,1) interval, assigned from internet-acquired knowledge, summing up to 1.0 for every place. $d_{T}$ is computed from (6) as

(6) $$d_{T}=P_{T}^{{}^{\prime }}.d$$

where d is the vector of distances d(i) for $i=1,2,\ldots ,N_{tp}$ given in Table A.6. For the ith place, d(i) is obtained using Google maps considered in the questionnaire. Finally, A is defined as

(7) $$A=\left[\begin{array}{c} e_{2}^{{}^{\prime }}A_{cpd}\\ A_{dir}\\ e_{1}^{{}^{\prime }}A_{cpd} \end{array}\right];\text{where}\;\;e_{1}\equiv \left[\begin{array}{c} 1\\ 0 \end{array}\right];e_{2}\equiv \left[\begin{array}{c} 0\\ 1 \end{array}\right]$$

**Fig. 9:**
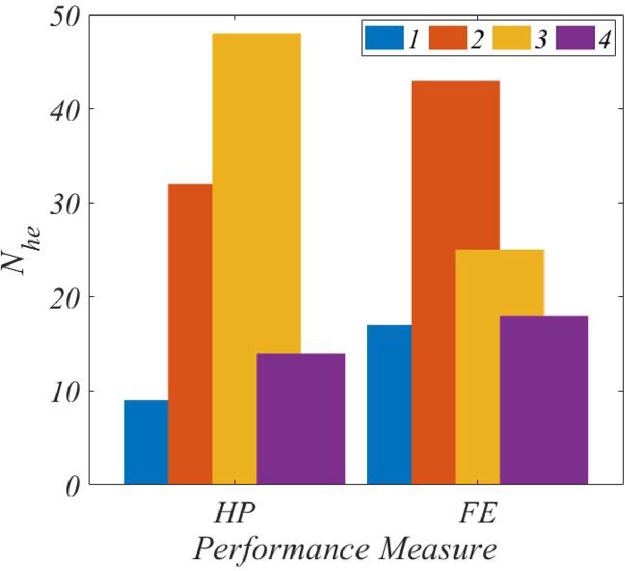
$N_{he}$ distributions, *PCP*.

Of 240 experts who were sent the questionnaire in Table A.2, opinion responses were received from 103 experts, which is thus the length of $C_{he}$. The frequency distribution of the responses to the questionnaire is shown in Fig. 9, in which the numbers of responses, $N_{he}$, are plotted for *HP* and *FE*. As in Fig. 5, Fig. 9 shows a “dominant-high-frequency” for both performance parameters. In case of *HP*, for instance, the rating of 3 is shared by as many as 48 (47%) experts, whereas 43 (42%) user experts share the rating of 2 for *FE*. For both distributions, the $\chi^{2}$ goodness-of-fit test confirms the “normal” character at a 5% significance level.

$C_{fes}:$Table A.4 gives the rules matrix used to produce each element of $C_{fes}$. Four memberships are adopted for each fuzzy variable: The consequent has E (excellent), VG (very good), G (good), and F (fair) as memberships, whereas the antecedents have different membership nomenclatures relevant to nine different background factors; however, all membership functions are Gaussian. Consequent memberships E, VG, G and F have peaks at x=1,2,3, and 4 in the support $\left(x_{\min},x_{\max}\right)\equiv \left(1,4\right),$ whereas antecedents have the support max. normalized to unity, $\left(x_{\min},x_{\max}\right)\equiv \left(0,1\right)$, with peaks of respective memberships located equidistantly within the support.

$C_{so}:$$C_{so}$ is derived by sampling from normal distributions as in the *IDP* domain. These distributions are associated with the four opinions E, VG, G and F in Table A.4 with the *centers of truth* located at x=1,2,3, and 4, respectively.

***Optimum rule weights:*** The *GA* runs adopted the same population size, crossover fraction, and convergence tolerance as for the *IDP*. The runs were made for a total of 11 increasing values of $N_{sr}=10,20,30,50,60,70,80,90,100,125$ and 150. On convergence, from the optimized W for each $N_{sr}$, the metric $W_{k}^{sr}$ is computed as defined by (3).

Table 2 gives the $W_{k}^{sr}$ for each of the rules discussed for the *PCP*. From Table 2, in case of *HP*, for instance, we see that rules 3 and 4 ($W_{k}^{sr}=1.0$ and 0.8222, respectively) are far more significant than the other rules.

**Table 2:** $W_{k}^{sr}$ vs. Rule k, *PCP*.

**No.**	**Performance parameter**	**Rule,**k							
		**1**	**2**	**3**	**4**	**5**	**6**	**7**	**8**
1	*HP*	0.0004	0.0969	1.0000	0.8222	0.5776	0.0009	0.4946	N/A
2	*FE*	0.0001	0.1453	0.0001	0.0001	1.0000	0.9907	0.5484	0.2846

**Fig. 10:**
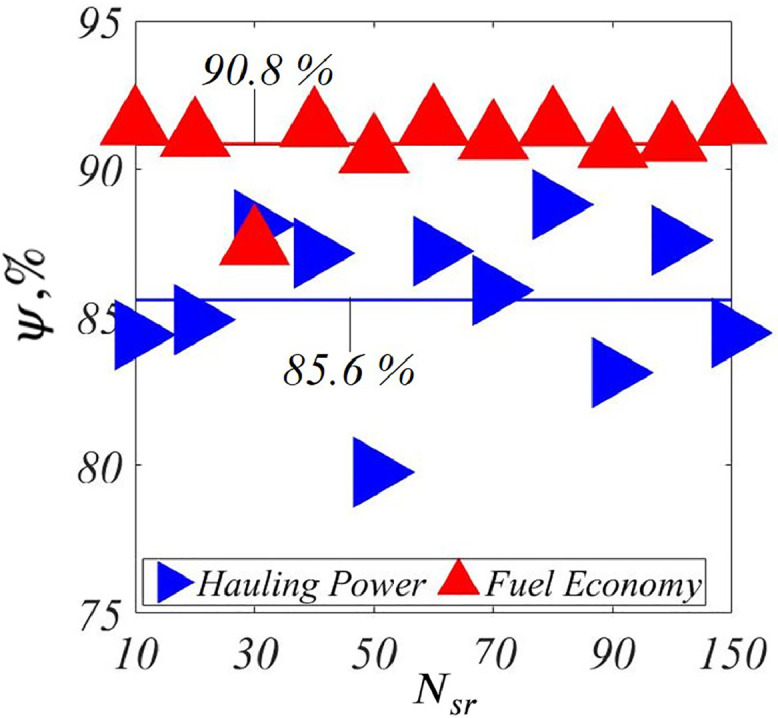
ψ vs. $N_{sr}$, *PCP*.

***Correlations,***ψ**:**Figure 10 shows the correlations, ψ, across $N_{sr}$ for both the performance parameters of *PCP*. We see that ψ is relatively high, going from ψ=85.6% to ψ=90.8% on an average, across the $N_{sr}$. This again demonstrates that MOSY is effective resulting in a high agreement between synthetic and expert user opinions.

***Convergence of***$t_{xp}$**:** For every $N_{sr}$ case, the CPU time $t_{opt}$ for *GA* optimization was recorded. From this time, we determined the average CPU time per expert opinion, $t_{xp}$, defined by (8)

(8) $$t_{xp}=\frac{t_{opt}}{N_{sr}}$$

**Fig. 11:**
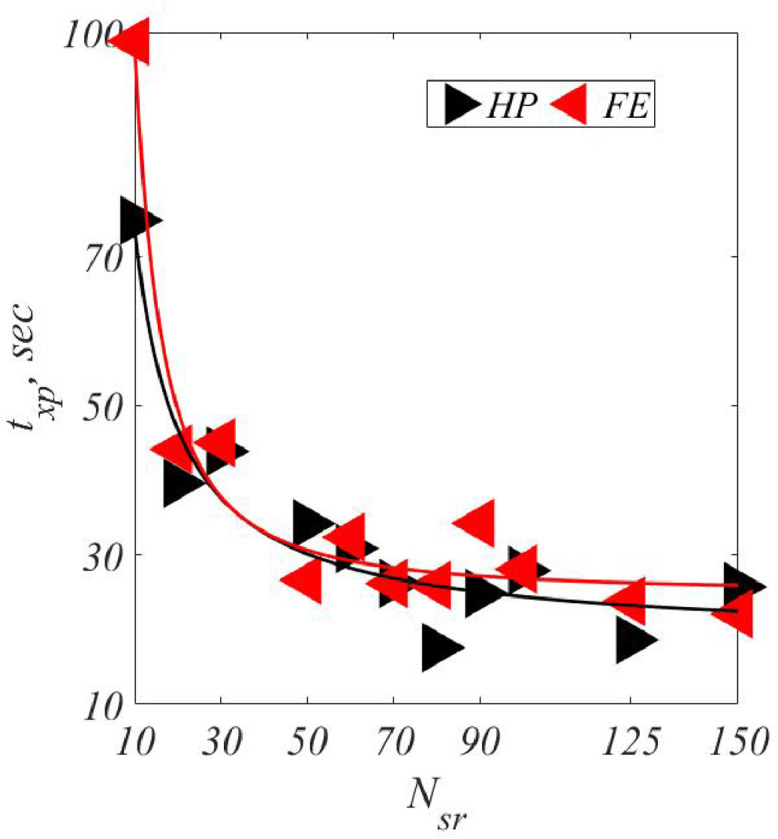
$t_{xp}$ vs. $N_{sr}$.

For both *HP* and *FE*, Fig. 11 shows the graphs of $t_{xp}$ vs. $N_{sr}$, including the best fit curves of three parameters using MATLAB curve-fitting toolbox. A clear convergence of $t_{xp}$ for both *HP* and *FE* is seen. Such convergence is noteworthy as it indicates **robustness of solution** and is reasoned as follows: precisely, with increasing $N_{sr}$, one could expect relatively more samples clustered *around* the centers of truth than *away* from them. On the other hand, the “signal-to-noise” ratio, in which (with respect to a center of truth) signal is “being near to”, while “being far from” is noise, would converge with increasing samples, $N_{sr}$. Indeed, $t_{xp}$ measures this ratio. Finally, from Fig. 11, we see that $N_{sr}=70$ would be a **minimum** to ensure robustness of the *FES*.

## Summary

In many domains, decision-making is challenging, as experts are often limited in availability. However, without a sufficient number of expert opinions, the associated solutions would not be robust. Motivated by this, MOSY was developed to produce a robust *FES* by specifying $N_{sr}$, the number of (synthetic) experts per rule.

In the framework of MOSY, corresponding to every fuzzy rule, antecedent vectors were sampled from uniform distributions as many as $N_{sr}$ times to produce as many human expert consequent opinions using the standard Mamdani inference process. Independently, corresponding to each fuzzy rule, as many synthetic consequent opinions were produced by sampling from normal-distributed memberships. The corresponding opinions-differences-vectors norm was minimized by optimization of the associated rule weights using a *GA*. MOSY was tested against sets of human expert opinions in two distinct domains, *IDP* and *PCP*. The results show that the synthetic and human expert opinions correlated between 91.4% and 98.0% on an average over $5\leq N_{sr}\leq 250$ across five outcomes of the *IDP*. Likewise, for the *PCP*, respective correlations varied between 85.6% and 90.8% for $10\leq N_{sr}\leq 150$ across two performance measures. These strong correlations lead us to conclude that MOSY is capable of producing synthetic opinions to yield a robust *FES* where sufficient human experts are not available.

## CRediT authorship contribution statement

**N. Gnaneshwara:** Conceptualization, Methodology, Software, Formal analysis, Resources, Writing – original draft. **B.V. Vijay:** Conceptualization, Methodology, Supervision, Writing – review & editing.

## Declaration of Competing Interest

The authors declare that they have no known competing financial interests or personal relationships that could have appeared to influence the work reported in this paper.

## Figures and Tables

**Table A.1:** Questionnaire, *IDP*.

**Ratings of background factors on project outcomes**	
**Background factor**	Answer 1,2,3,4 OR 5 ONLY*
Timely Approval of funds	
Management’s involvement	
Fair and transparent communication	
Resolution of conflicts	
Environment for innovative ideas	
Learnability	
Technical knowledge	
Technical Diversity	
Working relationship	
Team’s passion	
Ease of access to knowledge	
Timely updates on project	
Ratings of outcome measures in projects	
**Outcome measure**	Answer 1,2,3,4 OR 5 ONLY*
Innovativeness	
Knowledge Capital	
Cost Adherence	
Schedule Adherence	
Quality of Deliverables	

⁎ 1 = very low impact and 5 = very high impact.

**Table A.2:** Questionnaire, *PCP*.

**Profile**	
Age	
Number of family members	
Places visited	
Place	Y/N
Ladakh	
Manali	
Kulu	
⋮	
Mysore	
Ratings of vehicle performance	

**Parameter**	Answer 1,2,3 OR 4 ONLY*
Hauling power	
Fuel economy	

⁎ 1 = fair and 4 = excellent.

**Table A.3:** Rules, *IDP*.

**No.**	**Background factor**	**Rule**																			
		**1**	**2**	**3**	**4**	**1**	**2**	**3**	**4**	**1**	**2**	**3**	**4**	**1**	**2**	**3**	**4**	**1**	**2**	**3**	**4**
1	Timely approvals of the funds	H	H	L	L													H	H	L	L
2	Management’s involvement	H	H	L	L					VH	H	L	VL	VH	H	L	VL	H	H	L	L
3	Fair and transparent communication													VH	H	L	VL				
4	Resolution of conflicts									VH	H	L	VL					VH	H	L	VL
5	Environment for innovative ideas	VH	H	L	VL													VH	H	L	VL
6	Learnability					VH	H	L	VL												
7	Technical knowledge	H	H	L	L					VH	H	L	VL					VH	H	L	VL
8	Technical diversity					H			L												
9	Working relationship									H	H	L	VL	VH	H	L	VL				
10	Team’s Passion	VH	H	L	VL																
11	Ease of access to knowledge					VH	H	L	VL	VH	H	L	VL								
12	Timely updates on project	H			L									VH	H	L	VL	H	H	L	L

**No.**	**Outcome measure**	**1**	**2**	**3**	**4**	**1**	**2**	**3**	**4**	**1**	**2**	**3**	**4**	**1**	**2**	**3**	**4**	**1**	**2**	**3**	**4**

1	Innovativeness	VH	H	L	VL																
2	Knowledge capital					VH	H	L	VL												
3	Cost adherence									VH	H	L	VL								
4	Schedule adherence													VH	H	L	VL				
5	Quality of deliverables																	VH	H	L	VL

**Table A.4:** Rules, *PCP*.

**No.**	**Background factor**	**Rule**														
		**1**	**2**	**3**	**4**	**5**	**6**	**7**	**1**	**2**	**3**	**4**	**5**	**6**	**7**	**8**
1	Distance travelled		VH		H		L	VL		VH		H		L	VL	
2	Family Size	VS	VB	S	B	VS	VB	VS	VS	VB	S	B	VS	VB	VS	VS
3	Age	VY	Y	Y	M	M	M	EL	VY	Y	Y	M	M	M	EL	VY
4	Adventurous place	VH		H		L		VL	VH		H		L		VL	VH
5	Heritage place					H		L					H		L	
6	Leisure place					H		L					H		L	
7	Scenic place			VH		H		VL			VH		H		VL	
8	Spiritual place			H		H		VL			H		H		VL	
9	Wildlife place	VH		H		L		VL	VH		H		L		VL	VH

**No**.	**Performance parameter**	**1**	**2**	**3**	**4**	**5**	**6**	**7**	**1**	**2**	**3**	**4**	**5**	**6**	**7**	**8**

1	Hauling power	E	E	VG	VG	G	G	F								
2	Fuel economy								E	E	VG	VG	G	G	F	F

**Table A.5:** Category-weights, $W_{ctg}\left(i,j\right)$.

i	Place	**Category,**j					
		**Spiritual**	**Adventurous**	**Scenic**	**Heritage**	**Wild life**	**Leisure**
1	Leh–Ladakh	0.1	0.6	0.3	0	0	0
2	Amarnath	0.6	0.3	0.1	0	0	0
3	Manali	0.1	0.2	0.35	0.1	0	0.25
4	Vaishnodevi	1	0	0	0	0	0
5	Sikkim	0.2	0	0.5	0	0	0.3
6	Mussorie	0.2	0	0.4	0.1	0	0.3
7	Nainital	0.1	0.1	0.4	0	0	0.4
8	Rishikesh	0.45	0.2	0.25	0	0.1	0
9	Shimla	0.1	0.1	0.35	0.1	0	0.35
10	Dalhousie	0	0.1	0.35	0.25	0	0.3
11	Kulu	0.4	0.1	0.25	0	0	0.25
12	Gangtok	0.2	0	0.5	0	0	0.3
13	Darjeeling	0	0	0.5	0.1	0	0.4
14	Corbett National park	0	0.2	0	0	0.8	0
15	Rann of Kutch	0	0.1	0.9	0	0	0
16	Agra	0	0	0	1	0	0
17	Binsar	0.1	0.1	0.45	0	0.35	0
18	MountAbu	0.4	0.1	0.4	0.1	0	0
19	Pushkar	0.8	0	0.2	0	0	0
20	Sundarbans National Park	0	0.2	0	0	0.8	0
21	Jaipur	0.2	0	0	0.8	0	0
22	Varanasi	0.75	0	0.1	0.15	0	0
23	Andaman and Nicobar Islands	0	0.25	0.25	0	0.3	0.2
24	Khajuraho	0.5	0	0	0.5	0	0
25	Ajantha and Ellora	0	0	0	1	0	0
26	Lavasa	0	0	1	0	0	0
27	Mahabaleshwar	0.2	0	0.6	0	0	0.2
28	Lashadweep Islands	0	0.2	0.4	0	0	0.4
29	Alleppey	0	0	0.4	0	0	0.6
30	Thekkady	0	0	0	0	0.3	0.7
31	Goa	0	0.1	0.15	0.15	0	0.6
32	Munnar	0	0	0.5	0	0	0.5
33	Dandeli	0	0.1	0	0	0.6	0.3
34	Kodaikanal	0	0	0.5	0	0	0.5
35	Mahabalipuram	0.5	0	0	0.5	0	0
36	Ooty	0	0	0.5	0	0	0.5
37	Wayanad	0	0	0.5	0	0	0.5
38	Yercaud	0	0	0.5	0	0	0.5
39	Chickmagalur	0.1	0.1	0.3	0.1	0.1	0.3
40	Coorg	0	0.15	0.3	0	0.25	0.3
41	Bandipur	0	0	0	0	0.8	0.2
42	Mysore	0.2	0	0.1	0.3	0.1	0.3

**Table A.6:** Distances* of places, d(i).

i	**Place**	d(i)
1	Leh-Ladakh	3400
2	Amarnath	2900
3	Manali	2900
4	Vaishnodevi	2800
5	Sikkim	2700
6	Mussorie	2650
7	Nainital	2600
8	Rishikesh	2600
9	Shimla	2600
10	Dalhousie	2530
11	Kulu	2500
12	Gangtok	2350
13	Darjeeling	2300
14	Corbett National park	2125
15	Rann of Kutch	2100
16	Agra	2000
17	Binsar	2000
18	MountAbu	2000
19	Pushkar	2000
20	Sundarbans National Park	2000
21	Jaipur	1805
22	Varanasi	1800
23	Andaman and Nicobar Islands	1600
24	Khajuraho	1553
25	Ajantha and Ellora	1000
26	Lavasa	950
27	Mahabaleshwar	850
28	Lashadweep Islands	700
29	Alleppey	600
30	Thekkady	600
31	Goa	550
32	Munnar	500
33	Dandeli	450
34	Kodaikanal	400
35	Mahabalipuram	350
36	Ooty	317
37	Wayanad	300
38	Yercaud	300
39	Chickmagalur	250
40	Coorg	250
41	Bandipur	210
42	Mysore	180

⁎ From Bangalore, km.

## Data Availability

Data will be made available on request.
